# The Occurrence of Legacy P Soils and Potential Mitigation Practices Using Activated Biochar

**DOI:** 10.3390/agronomy11071289

**Published:** 2021-06-25

**Authors:** Vasile Cerven, Jeff M. Novak, Ariel A. Szögi, Kenneth Pantuck, Don W. Watts, Mark G. Johnson

**Affiliations:** 1 Water and Plant Research Center, Coastal Plains Soil, Agricultural Research Service, United States Department of Agriculture, 2611 W. Lucas Street, Florence, SC 29501, USA; 2 State Assistance & Partnerships Branch Infrastructure and Assistance Section, Water Division, U.S. Environmental Protection Agency, Philadelphia, PA 19103, USA; 3 Center for Public Health and Environmental Assessment, Pacific Ecological Systems Division, U.S. Environmental Protection Agency, Corvallis, OR 97333, USA

**Keywords:** soils, legacy P soils, activated biochar

## Abstract

The long-term application of manures in watersheds with dense animal production has increased soil phosphorus (P) concentration, exceeding plant and soil assimilative capacities. The P accumulated in soils that are heavily manured and contain excess extractable soil P concentrations is known as legacy P. Runoff and leaching can transport legacy P to ground water and surface water bodies, contributing to water quality impairment and environmental pollution, such as eutrophication. This review article analyzes and discusses current and innovative management practices for soil legacy P. Specifically, we address the use of biochar as an emerging novel technology that reduces P movement and bioavailability in legacy P soils. We illustrate that properties of biochar can be affected by pyrolysis temperature and by various activating chemical compounds and by-products. Our approach consists of engineering biochars, using an activation process on poultry litter feedstock before pyrolysis to enhance the binding or precipitation of legacy P. Finally, this review article describes previous examples of biochar activation and offers new approaches to the production of biochars with enhanced P sorption capabilities.

## 1. Introduction

The intensification of agricultural production through inorganic and organic fertilizers leads to crop yield increase and productivity in higher scale livestock operations. Although these agricultural intensifications are increasingly benefiting sustainable food production and security, they also increase concerns about the deterioration of environmental quality, soil nutrient balance, and crop productivity. This environmental concern is significant when agricultural waste products are used as a soil amendment, particularly in fields close to watersheds with intensive livestock production operations. For example, the intensive animal production system in the Chesapeake Bay Watershed area generates about 36 million metric tons of livestock manure per year [1].

**Figure 1.**
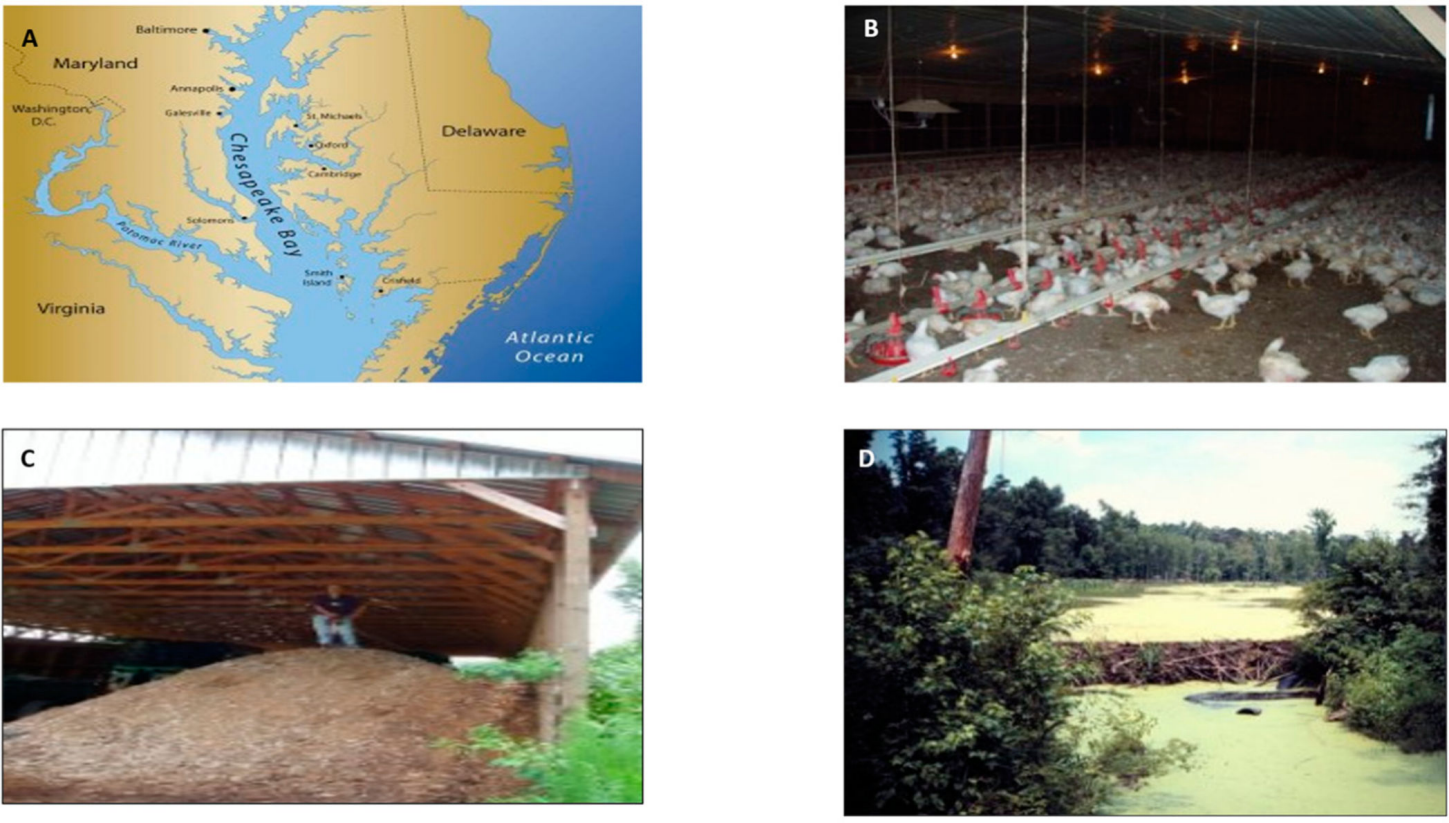
Chesapeake Bay region (**A**) containing high poultry populations (**B**), producing mounds of poultry litter manure (**C**), and a eutrophic wetland (**D**) from elevated stream water phosphorus concentrations (photos courtesy of Ariel Szögi and Jeff Novak, USDA-ARS).

Manure, (Figure 1) especially from poultry, is a leading source of P pollution in Chesapeake Bay [2], containing on average 32.6 kg ton^–1^ of total N and 31.3–35.4 kg ton^–1^ P_2_O_5_ of chicken litter [3,4]. A long-term trend for P accumulation was observed in topsoil (0–30 cm) over 12 years, as reported by [5]. In their study, P from poultry manure accumulated beyond plant nutritional requirements because the manure was applied at the agronomic N-recommended rates in a corn–soybean rotation system.

While livestock manure is a valuable resource for sustainable soil management and the supply of plant nutrients (e.g., N, nitrogen; P; and K, potassium), implementation of appropriate livestock manure management protocols is crucial for sustainable crop production and environmental quality. The need for manure guidelines becomes apparent when it is indiscreetly applied to soil as an organic fertilizer in small geographic areas with intensive animal production systems. It has been reported that manure application based on plant N nutrient requirements accumulates heavy metals [6,7] and excess soil P [8,9]. Current manure management practices based on crop N uptake requirements are unsustainable because of high risks of water quality impairment and soil nutrient and heavy metal concentration imbalances.

Although the agronomic soil P test is extensively used for making soil fertility recommendations, the soil P storage capacity (SPSC) concept has been proposed as a diagnostic tool for the assessment of the potential of P movement from the field environment to surface waters; SPSC can also be used to estimate legacy P loss at excessive concent [10] rations for soils in the eastern and central parts of the United States. The SPSC is based on a threshold molar ratio of extractable P/(Al + Fe) called the soil P saturation ratio (PSR), above which water soluble P abruptly increases. The research results revealed that [11] the degree of P saturation (DPS), which relates a measure of P already adsorbed by the soil to its adsorption capacity, could be an indicator of the soil’s P release capability. Particularly, the threshold DPS (Mehlich-1 extract) of 30% is recommended for Florida sands, while DPS values of 31–60% warrant caution with regard to the further addition of P to land, and DPS values of >60% indicate soils as contributors to water quality impairment [12]. As a result, P stability in soil with values below the threshold indicate that P release from the soil is minimal [13]. Kleinman and Sharpley’s [14] research results showed that Mehlich-3 extractable P can effectively be used to estimate P sorption saturation for over a wide range of acidic and alkaline soils as well.

Research results revealed that the long-term application of manure above plant nutritional requirements led to soil P accumulation, which is a potential source of environmental pollution [15]. For example, Sharpley et al. [16] reported higher water-extractable (11–85 mg kg^−1^) and Mehlich-3 extractable P concentrations (82–2840 mg kg^−1^) in 0–5 cm of soils following 10–25 years of manure (dairy, poultry, and swine) applications, as compared to water-extractable P (0.6–6 mg kg^−1^) and Mehlich-3 extractable P (4.0–64 mg kg^−1^) concentrations in untreated soils. The increase in both water- and Mehlich-3 extractable P in the heavily manured soil is 18–14 and 20–44-fold, respectively, higher than that for soil without manure application. Indeed, applied manure N and P are higher than the soil’s assimilative capacity and plant’s nutrient needs. This condition can result in promoting quality declines in water resources through soil leaching and runoff [17,18].

Phosphorus movement and accumulation in soils have been under investigation for several decades [19]. Novak et al. [17] reported P accumulation in soil and movement to a greater soil depth (60–152 cm) beneath a spray field that applied swine manure to a grass crop. These researchers speculated that severe P leaching was due to an exceedance of the soil’s profile P sorption capacity. Other researchers reported similar findings where the intensive over-application of swine manure to the same field might have overwhelmed the P sorptive capacity of Coastal Plain soils, which subsequently affected shallow groundwater quality [11,20]. Additionally, storm events will exacerbate water quality impairment linked to soil P buildup by increasing P runoff into waterways, leading to eutrophication and negatively impacting water quality [21,22]. Therefore, the development of a new management strategy to eliminate legacy P losses from P-overloaded soils is vital for environmental sustainability. Moreover, P as a resource is needed since global P supplies are not infinite [23].

## 2. Management Practices That Reduce Agricultural Losses of Legacy P

It is important to develop the best agronomic management practices capable of controlling legacy P losses in agricultural soils, especially its dissolved forms. The overall goal is to minimize P movement and not to create water quality impairment that exacerbates environmental pollution. These practices intend to reduce legacy P leaching as one of the main pathways of its loss, largely in sandy soils in the Southeastern USA, which have limited soil phosphorus storage capacity and high infiltration rates [11,24]. As the research results have revealed [17,25], the long-term application of animal manure caused strong accumulation and the downward movement of dissolved P through the soil profile. Phosphorus movement through the soil profile also presents threats to water quality. For example [26], reported that during storms, the rain infiltrating high P topsoil mobilized the dissolved P into groundwater, causing considerable P movement as deep as 1.5 m. Similarly, Kleinman et al. [27] also observed that overland flow from fields to ditches accounted for about 8% of annual ditch P export, and that more than 90% of P transport from fine-textured Delmarva soils to field ditches occurred in subsurface flow. Therefore, controlling dissolved P losses from agricultural soils to surface waters is challenging because conservation practices mostly prevent (particulate) soil P losses.

### 2.1. Agronomic Practices

Here we describe management practices that reduce legacy soil P, which is vital to the minimization of P losses from agricultural soils, and those that mitigate the risk of eutrophication. A variety of agronomic practices exist to reduce legacy P concentrations and movement from agricultural soils. These agronomic practices target the P transport pathway and involve the implementation of soil and nutrient management practices [28]. Several agronomic practices addressing legacy P losses include conservation tillage [29,30] and cover crops [31,32]. These practices create vegetative buffer strips along water bodies [33,34]. The implementation of these buffer strips also includes grass–legume forage systems [35] combined with the best nutrient management efforts such as the land application of P-sorbing materials; treating manure with aluminum sulfate; increasing plant P use efficiencies; and increasing the N:P ratio of manure [36,37]. However, these best management practices can be more effective on sediment-bound P in the runoff, but less effective in reducing dissolved P loss from sandy soils on the Delmarva Peninsula [38,39].

### 2.2. P-Hyperaccumulator Plants

An emerging technology that ameliorates the legacy P issue is phytoremediation. This technology involves the use of plants for the uptake of pollutants from the environment. Several plant species such as Indian mustard (*Brassica juncea* L.), corn (*Zea mays* L.), and annual ryegrass (*Lolium multiflorum*) grown on a highly phosphorus-enriched soil exhibited higher P uptake and can thus be employed for P phytoremediation [40,41]. White lupin (*Lupinus albus* L.) responded to the level of soil residual P in the Coastal Plain of South Alabama and can hyperaccumulate P [42]. Indeed, Novak and Chan [43] proposed to reduce higher concentrations of soil P in the fields with excess P levels by using P-hyperaccumulator plants or by growing plants that are genetically modified through traditional breeding and transgenic technique strategies in order to increase their P-uptake characteristics. A recent study revealed that P adsorption in transgenic plants increased 3-fold as compared to that in host plants (Torenia hybrid cv. Summer wave blue; Petunia hybrid cv. Surfinia purple mini; and Verbena hybrid cv. Temari scarlet), according to [44]. The transgenic plants showed hyperaccumulation of inorganic P in their leaves and accelerated their absorption rates in hydroponic solutions.

### 2.3. P-Binding Technologies

#### 2.3.1. P-Sorbing Materials

P-sorbing materials offer a new approach to the reduction of dissolved P concentration and its movement. Natural and industrial by-products commonly contain chemical compounds such as iron (Fe), aluminum (Al), calcium (Ca), and magnesium (Mg) oxides or oxyhydroxides. P-sorbing materials have been applied as a soil amendment at rates of 2.5%, 5.0%, 7.5%, and 10% (*w*/*w*) [45]. These compounds can potentially chemically bind P through various sorption mechanisms or through the formation of insoluble precipitation phases [46,47]. For example, the removal efficiencies for cumulative P from swine wastewater using the marl gravel media filter system ranged from 37% to 52% [48]. Another industrial by-product—the acid mine drainage (AMD) treatment residuals—was found to decrease plant available P in poultry litter [49], in soil [50,51], as well as when used in a drainage ditch [52]. Water treatment residuals (WTR), a by-product of municipal drinking water treatments plants, have a strong affinity to sorb P as well [53], and its adsorption capacity can vary depending on WTR application rates [54]. Research results obtained using P-enriched Coastal Plain sandy soils revealed [45] that applying alum-based WTR into an Autryville and Norfolk soil series significantly increased their Pmax values relative to soils with no WTR addition. Furthermore, the results showed that WTR incorporation into soils with high P concentrations caused larger relative reductions in extractable water soluble P than Mehlich-3 P concentrations [55]. Moreover, coarse-sized WTR aggregates (between 1.0 and >4.0 mm) showed less adsorption capacity than fine-sized (<1.0 mm) aggregates [56]. The addition of Fe/Mn- and P-modified Al-WTR to the soil significantly reduced the concentrations of Pb (up to 60% by Fe/Mn-Al-WTR and 32% by P-Al-WTR) and Cu (up to 45% by Fe/Mn-Al-WTR and 18% by P-Al-WTR) in the shoots and roots of ryegrass as compared to raw Al-WTRs and untreated soil, according to [57].

#### 2.3.2. Naturally Occurring and Waste Materials

It appears that the use of gypsum is a valuable strategy for controlling P movement [58,59]; likewise, there is a large potential in the use of waste materials such as bauxite residuals, fly-ash, wood ash, and slag [52]. Pen et al. [60] observed that using steel slag as the P sorption material in the P removal structure resulted in the removal of 25% of all dissolved P from rainfall and irrigation events during the first five months of structure operation. The other study demonstrated that the maximum adsorption capacity of the fly-ash and bauxite residuals were 29 and 25 g kg^−1^, respectively [61], and P removal varied based on the chemical properties of the by-products [62,63]. Therefore, P mobility can be controlled by Al, Fe, or Ca depending on their pH. As an example, the P removal by AMD residuals and WTRs was a result of the adsorption to Al- or Fe-oxides/hydroxides or the precipitation of Al- or Fe-phosphates. In contrast, P removal from stormwater by slag materials used in field scale filtration structures occurred through both Ca and Al/Fe mechanisms [64]. The mobility of P is also highly pH dependent. Lee et al. [65] observed that a higher P adsorption rate onto WTR was obtained at low reaction media (pH 4) as compared to neutral media, with the lowest P adsorption rate at pH 9. As Silva [66] reported, the two highest peaks occur in the soil pH acid range of pH 4–5.5, where P precipitates with Fe and Al, while the third peak occurs in alkaline soils at around pH 8.0, when P is precipitated primarily by Ca.

### 2.4. Biochar

Among the several chemical/physical methods developed to reduce legacy soil P [55,56], biochar emerges as a novel technology for the binding of P-forms [67,68]. Biochar is the carbonaceous by-product from the thermochemical conversion of organic materials that commonly contain high amounts of cellulose, hemicellulose, or lignin [69]. Kang et al. [70] observed that biochar application improved the soil bulk density, soil organic carbon, pH, and cation exchange capacity in field soil, which positively affected corn and Chinese cabbage growth. Biochar can be produced from a wide range of biomass feedstocks including woodchips from cedar, cypress, bamboo, pine sources [71,72], agricultural by-products, or residues of agricultural wastes including pecan shells, peanut shells, cotton gin trash, wheat straw, corn straw, corn cobs, rice husk [73,74], and livestock manure [75,76].

The pyrolysis temperature for biochar derived from poultry litter, cattle manure, rice straw, soybean straw, and corn was 450 °C [76]; the temperatures from peanut hull were 400, 500 °C; those from pecan shell were 350, 700 °C; those from poultry litter were 350, 700 °C; those from switchgrass were 250, 500 °C [73]; that from pine chips, poultry litter was 500 °C [75]; and those from wood chips: Japanese cedar, Japanese cypress, bamboo chips, rice husks, sugarcane bagasse, and poultry manure were 400, 600, and 800 °C [71]. The choice of feedstock for the production of biochar is often decided by local availability of waste produce and transport distance to the pyrolyzer plant [77,78]. After pyrolysis, biochar properties are often modified physically [79,80] or chemically [81,82] to increase its surface area, pore size distribution, and form surface functional groups to increase the biochar’s adsorption capacity [83,84]. Physical biochar engineering techniques include ball milling modification, gas/steam activation, magnetization, and microwave irradiation [85]. Recent research results showed the potential for improving biochar P adsorption capacities by chemical modification [86,87], which includes its activation by different acids (HCI, HNO_3_, H_2_SO_4_, H_3_PO_4_ and H_2_O_2_), with different alkali (NaOH, KOH), with other oxidizing agents (KMnO_4_, Fe(III)) [85] and salts (MgCl_2_, CaCl_2_) [82,87,88]. Although recent studies and review papers [75,89] describe biochar’s properties and its role in soil remediation, the efficacy of activated biochar on soil legacy P remediation requires further investigation due to diverse feedstock chemistry [69], activation technologies [90,91], and the complexity of biochar structural properties as well as soil properties [92,93].

We compiled a list of published works that used various chemical/physical activation processes on biochar produced from various feedstocks and how they responded in P sorption experiments. Results in Table 1 reveal that the P adsorption capacity among the biochars varied considerably. We report that from these studies, the Pmax values ranged from 13.6 to 153.4 mg^−1^.

The studies cited in Table 1 used a number of chemical and physical activation processes on the biochars to improve their physicochemical properties and enhance their adsorption performance. Generally, chemically activating these biochars with these salts resulted in the biochar’s higher P adsorption capacity. MgO-biochar showed better phosphate adsorption in saline soils and the maximum phosphate adsorption capacity was 1.46 times higher than biochar [94]. Phosphates were bound to the Mg-biochar not only by electrostatic adsorption but also by covalent bonds to form magnesium phosphate crystals [88,94]. Moreover, as observed by [83], the P removal efficiency increased with the increasing adsorption dosage. Zheng et al. [96] reported that P was adsorbed by the Mg-Al modified biochar through co-precipitation reaction and an Al-designed biochar showed the highest aqueous stability with little metal dissolution. This designed biochar showed an increase in P adsorption with an increase in metal loading. Additionally, physical activation by modifying feedstock pyrolysis conditions (e.g., temperature, residence time, etc.) resulted in an increasing biochar adsorption capacity with higher pyrolysis temperatures [87,99], which varied across different feedstocks [95].

On a wider scale of use, our proposed concept of “designer” or engineered biochar may provide the means of making biochar to specifically address a targeted contaminant or ameliorate soil deficiency. It was suggested that biochar properties can also be modified to remediate problem soils [73]. For example, water holding capacity limitations of sandy soils can be improved with biochar made from pine chips, switchgrass made at 350 °C or miscanthus at 459 °C [100,101]. Similarly, [102] reported that date palm-derived biochar produced at low pyrolysis temperatures (300 °C and 400 °C) improved water retention in sandy soil by 46%. Moreover, biochar with particle sizes <1 mm can increase water conservation in sandy soil more than larger (1–2 mm) particle sizes [103]. As a result, designer biochar applied to nutrient-poor soils have been reported to increase soil fertility properties [104–106] and improve crop growth [107–109].

## 3. Conclusions and Prospects

Our review article summarizes recent research results using agronomic, physical, and chemical methods as best management practices in soil legacy P reduction. The review highlights the significant inter-relations between biochar properties vs. feedstock source, role of pyrolysis temperature, choice of activation chemical materials, and examples of using designer biochar to decrease soil legacy P concentrations. Additionally, we showed that designer biochar can be produced by chemically activating its parent feedstock with metal salts or other amendments, or through physical processes such as pyrolysis temperature modifications. These last two physico-chemical approaches produce biochar with enhanced P sorption capabilities and serve as alternative management practices in the reduction of legacy P soils. The overall benefit is the reduction of both extractable P concentrations and dissolved P concentrations in legacy P soils with the subsequent benefit of rebalancing soil P levels and reducing non-point source P pollution. Furthermore, biochar can be used as a soil amendment to improve soil quality by reducing the presence of heavy metals in contaminated soils, and as a slow-release fertilizer for improving the fertility of the agricultural soils. However, we suggest that further biochar research needs to adjust biochar application rates under field environment conditions. In this way, high-risk pollution from heavy metals contained in those by-products will be reduced.

## Figures and Tables

**Table 1.** Phosphorus (P) adsorption capacity of activated biochars derived from various feedstocks.

Feedstock	P Form	Activation	Carbonization	Adsorption	References
		Agents	Activation	Capacity	
			Conditions	mg g^−1^	
			(°C)	qmax	
Peanut shells	P	1 M MgCI_2_	600	18.9	[94]
Poplar chips	PO4^−3^	4% MgCI_2_	600	89.9	[87]
Soybean straw	PO4^−3^	2 M MgCI_2_	500	74.5	[82]
Ground coffee					
waste	P	3 M MgCI_2_	500	56	[88]
Sewage sludge+					
dolomite, 1:1	PO4^−3^	Dolomite	800	29.2	[83]
Banana straw	P	1 M MgCI_2_	430	31.2	[95]
Wheat straw	PO4^−3^	0.5 M MgCI_2_+			
		0.5 M AICI_3_	600	153.4	[96]
Dairy manure	PO4^−3^	2 M CaCI_2_	500	13.6	[97]
Crop residuals	P	3.1 M MgCI_2_	600	65.4	[98]
Tea residuals	PO4^−3^	0.001 M AgNO_3_	400	13.6	[99]

## Data Availability

Not Applicable.
